# Prevalence and Risk Factors of Chronic Obstructive Pulmonary Disease in Bangladesh: A Systematic Review

**DOI:** 10.7759/cureus.3970

**Published:** 2019-01-28

**Authors:** Ipsita Sutradhar, Rajat Das Gupta, Mehedi Hasan, Amit Wazib, Malabika Sarker

**Affiliations:** 1 Epidemiology and Public Health, Brac James P Grant School of Public Health, BRAC University, Dhaka, BGD; 2 Internal Medicine, Shahabuddin Medical College and Hospital, Dhaka, BGD

**Keywords:** prevalence, risk factors, chronic obstructive pulmonary disease (copd), bangladesh

## Abstract

Chronic obstructive pulmonary disease (COPD) is a leading cause of mortality and morbidity in low- and middle-income countries (LMICs) including Bangladesh. But no systematic review has been carried out in Bangladesh, which portraits the burden of COPD and its risk factors. Therefore, this systematic review was conducted to find out the prevalence and risk factors of COPD in Bangladesh. We searched PubMed, Google Scholar, Popline, and Banglajol from January 1, 1972 to April 30, 2017. We included studies that reported the prevalence and/or risk factors of COPD among Bangladeshi people. Two researchers independently searched and screened all the articles and extracted data from nine eligible studies. The whole process was verified by another researcher. Quality assessment was performed using a checklist adopted from published articles on quality assessment guidelines of observational studies. Discrepancies were resolved by consensus. Data analysis was done thematically. The pooled COPD prevalence among Bangladeshi adult was 12.5% (95% CI, 10.9-14.1) using the Global Initiative for Chronic Obstructive Lung Disease (GOLD) criteria and 11.9% (95% CI, 11.4-13.6) using the lower limit of the normality (LLN) criteria. The prevalence was higher among males, low socio-economic group, rural residents, and biomass fuel users. Tobacco consumption, exposure to biomass fuel, old age, and history of asthma were identified as major risk factors of COPD. COPD prevalence is high in Bangladesh. It is a timely need for the policy-makers and public health professionals to take pertinent steps for prevention and control of COPD in Bangladesh.

## Introduction and background

Chronic obstructive pulmonary disease (COPD) is defined by the American Thoracic Society (ATS) as a progressive and partially reversible disease of respiratory tract featured by the limitation of airflow that takes place as a result of chronic bronchitis or emphysema [1]. There are several established risk factors of COPD, of which smoking is the strongest one [2]. Indoor air pollution, occupational exposures to coal dust, silica and asbestos, low birth weight, and recurrent infections are also considered as risk factors of COPD [3].

COPD is a global public health concern of the 21st century. In 2005, worldwide, 5% deaths were attributable to COPD [4]. According to the Global Burden of Disease 2015, the mortality rate due to COPD increased almost by 11% from 1990 to 2015. During the same time period, the prevalence of the disease also increased by 44% [5]. If this current rate continues, COPD will be the third leading cause of global death by 2030 [6]. Remarkably, almost all (90%) of the deaths caused by COPD occurred in low and middle-income countries (LMICs) [7]. Bangladesh, like other LMICs, is facing epidemiological transition with an increasing burden of non-communicable diseases (NCDs) like COPD [8-9].

In Bangladesh, a number of studies have been conducted focusing on two major NCDs, namely diabetes and hypertension [10-12]. But limited data are available on COPD. Since 2000, several studies have been conducted in Bangladesh to identify the burden and risk factors of COPD. However, these studies differ significantly in terms of methodology (hospital-based and population-based study), sample size, and area of the study (urban/rural). Furthermore, different criteria were used to measure the COPD status in these studies. However, no systematic review has been carried out in Bangladesh to provide a clear scenario of COPD burden and risk factors in this country. Therefore, we carried out this systematic review with the aim to find out the prevalence of COPD and its associated risk factors among the Bangladeshi population. This evidence will help policy-makers to develop suitable policies and the public health professionals to design and implement interventions for prevention and control of COPD.

## Review

Materials and methods

Search Strategy

We systematically searched international (PubMed, Google Scholar, Popline) as well as national (Banglajol) databases to identify all potential publications on COPD in Bangladesh. We used both medical sub-headings and plain text for the following keywords: “epidemiology”, “prevalence”, “burden”, “risk factors”, “etiology”, “causality”, “aetiology”, “chronic obstructive pulmonary disease”, “COPD”, “pulmonary emphysema”, “emphysema”, “chronic bronchitis”, “bronchitis”, “obstructive lung disease”, “Bangladesh”. Using these keywords along with a Boolean operator, we prepared the global search term to identify the relevant publications. The team also manually searched the bibliography of initially selected articles (snowballing) to identify more studies related to COPD. We used ‘Preferred Reporting Items for Systematic Review and Meta-Analysis’ (PRISMA) guideline for reporting this systematic review [13].

Inclusion and Exclusion Criteria

This systematic review included published articles based on the following criteria: a) study reported data from Bangladesh; b) study published between 1st January 1972 (Bangladesh became a sovereign country on December 16, 1971) and 30th April 2017; c) study reported prevalence of COPD or emphysema or chronic bronchitis or obstructive lung disease; d) study reported single or multiple risk factors of COPD or emphysema or chronic bronchitis or obstructive lung disease; e) quantitative study; f) study published in the English language. However, studies were excluded based on the following criteria: a) study did not report data from Bangladesh; b) study published in other languages than English; c) qualitative study; and d) conference proceedings, book chapters, and editorials. At first, two researchers searched and screened all the articles individually. Another researcher critically reviewed the overall search and screening process to ensure the consistency. Finally, the full text of selected publications was assessed for eligibility by all three researchers. During the initial screening, it was not clear whether studies that focused on COPD or other respiratory diseases were included. However, while assessing the full text of these articles, any study that did not focus on COPD or obstructive lung disease was excluded. Any discrepancies during this process were resolved by group consensus.

Quality Appraisal

Three researchers independently determined the risk of bias of included studies. This study used a quality assessment checklist to assess the quality of the included studies. This checklist was prepared based on the criteria described in ‘Strengthening the Reporting of Observational studies in Epidemiology (STROBE)’ and other published methodological studies [14-16]. The criteria were:

1. Whether the study objective was clearly defined?

2. Whether the sampling technique was random or non-random?

3. Whether adequate sample size was used based on sample size calculation?

4. Whether COPD was measured objectively?

5. Whether COPD was measured based on any international criteria?

If the study objective was clearly defined, we scored it as “1”, otherwise “0”. If the included study used random sampling, we scored it as “1”; however, if the included study used non-random sampling or sampling technique was not clearly mentioned, we scored it as “0”. If the sample size calculation was clearly described and required sample was obtained or, if at least 384 samples were obtained, we scored it as “1”, otherwise “0”. If the researchers measured COPD objectively (using spirometry), we scored it as “1”, but, if they measured COPD subjectively (self-reported or symptom-based) or did not clearly mention how COPD was diagnosed, we scored it as “0”. If they measured COPD based on the Global Initiative for Chronic Obstructive Lung Disease (GOLD) criteria, we scored it as “2”; if they used any other international criteria, we scored it as “1”; and if it did not mention any criteria, we scored it as “0”. Later, the number for each study was added to obtain the final score. The maximum score was 6. If any study gets 6, we considered it as “high quality” study, scores 4 and 5 indicate “moderate quality”, and the lowest scores 0, 1, 2, and 3 indicate that the study was of "poor quality". All the discrepancies that arose during the quality assessment process were solved by the group consensus.

Data Extraction

A data extraction form was created in excel to conduct this review. This form included (a) title, (b) journal name, (c) name of authors, (d) publication year, (e) year of data collection, (f) study objective, (g) study setting (urban/rural), (h) study design, (i) sampling strategy (random/non-random), (j) sample size, (k) study population, (l) outcome assessment (objective/subjective), (m), diagnostic criteria for COPD, (n) prevalence (overall), (o) prevalence (gender, age, location specific), (p) risk factors, (q) authors’ conclusion, and (r) researchers’ observation. Two researchers independently extracted data using the data extraction form. Another researcher cross-checked and critically reviewed both the files extracted by the individual researcher. Any forms of dispute arose during the data extraction process were resolved by the group consensus. Data analysis was done thematically.

Results

Search Result

**Figure 1:**
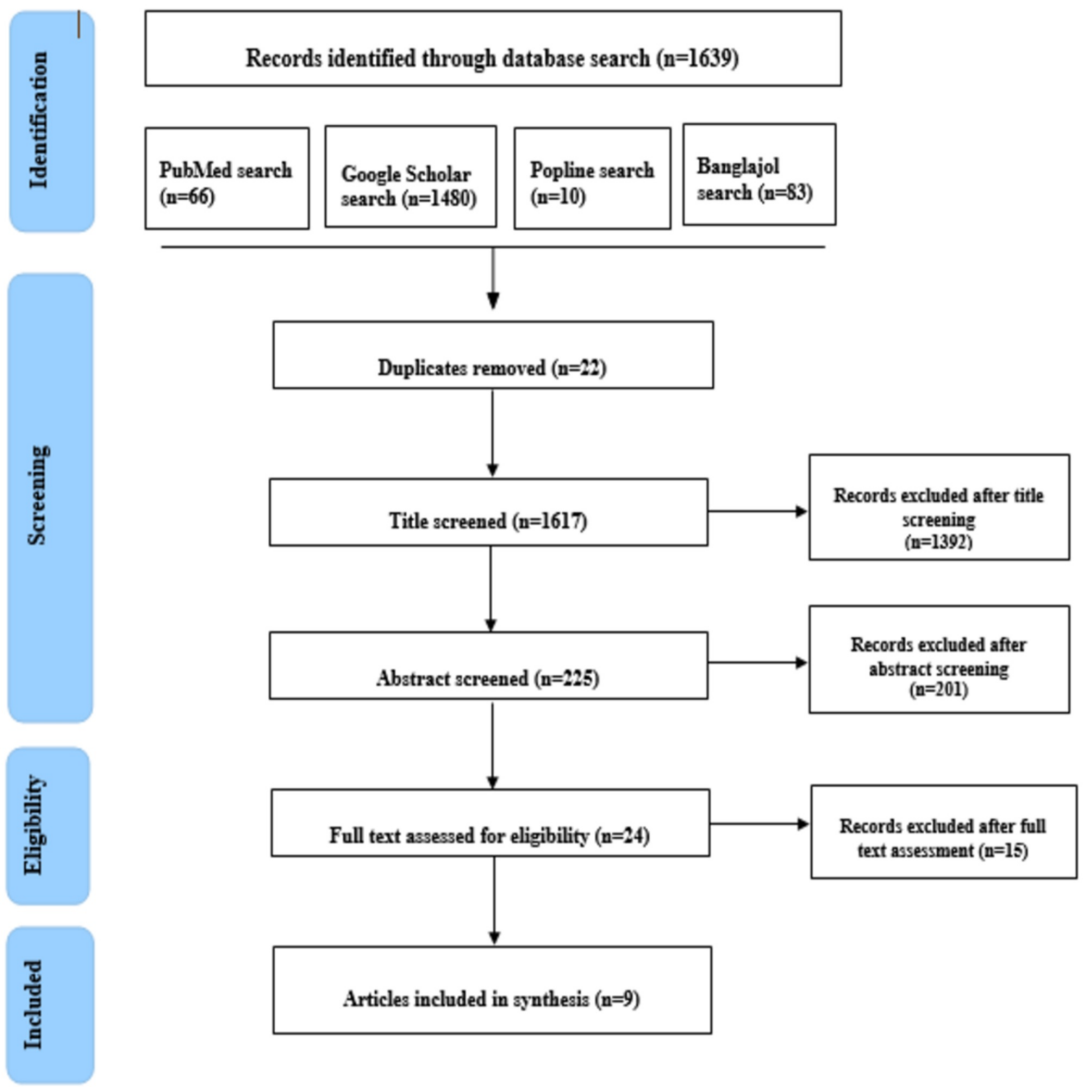
Flowchart showing steps of selecting articles

Initially, 1,639 articles were identified through a database search. Following duplicate removal, title and abstract screening, 24 articles remained for full-text assessment. The full text of these 24 studies was critically reviewed, of which nine studies met the eligibility criteria. Therefore, finally, nine studies were included in this systematic review. The overall process was done using the PRISMA flow diagram (Figure 1).

Study Criteria

All of the nine eligible studies were published in 2013 and onwards. Five of these studies were published in international, peer-reviewed journals [17-21] and three were published in national journals [22-24]. Of these studies, three included respondents from both urban and rural areas [17-19]. However, four studies were conducted among the urban population [20-21,24-25] and two studies involved participants only from rural settings [22-23]. Seven studies adopted a cross-sectional study design to identify the prevalence and risk factors of COPD [17,19-24]. One study performed secondary data analysis of population-based studies in 10 LMICs including Bangladesh [18]. The sample size of the included studies ranged between 108 and 3744 individuals. Six out of all nine studies collected data from both men and women [17-18,20,23-25]. The minimum age requirement was 40 years for three studies [17-18,22], 15 years for two studies [19,25], and 35 years for the remaining one study [24]. However, the maximum age limit was specified in only one study that was 65 years [25]. COPD alone was the outcome variable in five of these studies [17-18,20,22,24], whereas rest of the studies focused on other diseases (asthma, restrictive and combined pulmonary disease, and NCDs) and symptoms along with COPD [19,21,23,25].

Quality of the Included Studies

Among the nine studies included in our systematic review, two were of high quality [17,24], four were of moderate quality [18-19,21-22], and the remaining three were of low quality [20,23,25] based on the preselected criteria described in ‘Quality appraisal’ section. Detail of study quality is illustrated in Table 1. Closer inspection of the Table 1 shows, six out of nine studies clearly defined study objectives [17-19,21-22,24] and five studies used random sampling technique [17-18,21-22,24]. Only one study described sample size calculation and their sample size was 98 although they finally collected data from 108 randomly selected study participants [21]. Five studies, however, did not explain sample size calculation, but their sample size was decent (583 to 3744) [17-18,20,23-24]. One study, however, included participants from all the households of a randomly selected study site; therefore, describing the sampling technique and sample size calculation was not obligatory for them [19]. For the diagnosis of COPD, two studies used the GOLD criteria [17,22,24], while three studies used internationally standard criteria other than GOLD [17-19,21]. According to the GOLD criteria, FEV1/FVC <70% during post-bronchodilator spirometry is considered as the presence of COPD [26]. Outcome assessment (COPD) was objective in five studies [17-18,21-22,24] (Table 1).

**Table 1:** Quality appraisal of selected studies based on study criteria and allocated points COPD - Chronic obstructive pulmonary disease, GOLD - Global Initiative for Chronic Obstructive Lung Disease

Authors	Objective	Sampling strategy (random or nonrandom)	Sample size calculation	Diagnostic criteria for COPD	Outcome assessment (objective or subjective)	Total Score
Alam et al., 2015 [17]	Clearly defined-1	Random-1	Point-1 (SS -3744)	International criteria-2 (GOLD and LLN)	Objective-1	6
Grigsby et al., 2016 [18]	Clearly defined-1	Random-1	Point-1 (SS- Dhaka-1, 878; Matlab-1, 846)	International criteria-1 (Not GOLD)	Objective-1	5
Alim et al., 2013 [19]	Clearly defined-1	Census-1	Point-1 (SS-420; Census, so sample size calculation was not needed)	International criteria-1 (Not GOLD)	Subjective-0	4
Kabir et al., 2016 [20]	Not Clearly defined-0	Non-random-0	Point-1 (SS -583)	Not mentioned-0	Not mentioned-0	1
Mahfuz et al., 2014 [21]	Clearly defined-1	Random-1	Point-1 (SS was clearly described, SS was 96 but finally study conducted on 108)	International criteria-1 (Not GOLD)	Objective-1	5
Biswas et al., 2016 [22]	Clearly defined-1	Random-1	No-0 (SS-276)	International criteria-2 (GOLD)	Objective-1	5
Rahman et al., 2017 [23]	Not Clearly defined-0	Non-random-0	Point-1 (SS -1203)	Self-reported-0	Subjective-0	1
Islam et al., 2013 [24]	Clearly defined-1	Random-1	Point-1 (SS -900)	International criteria-2 (GOLD)	Objective-1	6
Ahmed, 2016 [25]	Not Clearly defined-0	Not mentioned-0	No-0 (SS -300)	Not mentioned-0	Not mentioned-0	0

Prevalence of COPD

To describe the prevalence of COPD, we considered data merely from high- and moderate-quality studies [17-19,21-22,24]. According to high-quality studies [17,24] that used the GOLD criteria, the pooled COPD prevalence among Bangladeshi adults was 12.5% (95% CI, 10.9-14.1) ranging between 11.4% (95% CI, 9.3-13.5), which was found among participants aged 35 years or more, and 13.5% (95% CI, 12.4–14.6), which was reported among participants aged 40 years or above (Table 2). However, using the lower limit of the normality (LLN) criteria, pooled COPD prevalence was 11.9% (95% CI, 11.4-13.6) ranging from 10.0% (95% CI, 8.6-11.4) in urban Dhaka to 15.4% (95% CI, 14.2-15.8) in rural Matlab [17-18]. Two moderate-quality studies were carried out on two specific groups of population: (a) women of rural Chittagong exposed to fuel smoke [22] and (b) helpers of human haulers of Dhaka city [21]. Remarkably, the prevalence of COPD was found much higher in these two groups of people (women exposed to fuel smoke: 20.4%; helpers of human haulers: 41.7%) [21-22].

**Table 2:** Prevalence and risk factors of COPD among Bangladeshi population COPD - Chronic obstructive pulmonary disease, GOLD - Global Initiative for Chronic Obstructive Lung Disease

Study	Study settings	Study design	Sample size	Study population	Outcome variable	Prevalence (%)	Distribution (%)	Risk factors
Alam et al., 2015 [17]	Rural-Matlab, Chandpur; Urban-Kamlapur, Dhaka	Cross-sectional observational study	3744 Urban-1895; Rural-1849)	Male and female of age >40 years	COPD	13.5 (95% CI, 12.4–14.6) (GOLD criteria) 10.3 (95% CI, 9.3–11.3) (LLN criteria)	Age Group 40–49 years- 5.2 50–59 years-13.6 60–69 years-27.5 Sex Male-22.0 Female-6.4 Area of residence Rural-17.0 Urban-9.9 Education Illiterate-16.1 Literate-11.3 Occupation Manual-24.9 Non-Manual-8.5 Income Low-16.3 High-11.1 BMI Normal- 12.4 Underweight- 26.8 Overweight/Obese- 5.9 Smoking Status Never- 6.1 Current- 24.8 Former- 28.1 Biomass Fuel Exposure Biomass Fuel- 17.3 Clean Fuel- 9.9 H/O of Asthma Yes- 38.5 No- 11.6 (According to GOLD criteria)	Age Group 50–59 years OR: 2.2 95% CI, 1.6–3.0 p < 0.001 60–69 years OR: 4.7 95% CI, 3.5–6.4 p < 0.001 Education (Illiterate) OR =1.4 95% CI, 1.1–1.7 p = 0.008 BMI (Underweight) OR: 1.9 95% CI, 1.5–2.4 p < 0.001 Smoking Status Current OR: 5.5 95% CI, 4.2–7.2 p < 0.001 Former OR: 4.5 95% CI, 3.3–6.0 p < 0.001 Biomass Fuel Exposure OR: 1.5 95% CI, 1.2–1.9 p = 0.001 H/O of Asthma OR: 6.9 95% CI, 4.9–9.5 p < 0.001
Grigsby et al., 2016 [18]	Ten LMICs; In Bangladesh: Rural-Matlab Urban-Dhaka	Longitudinal study (Bangladesh)	Dhaka-1878; Matlab-1846	Male and female of age > 40 years (Bangladesh)	COPD	Dhaka- 10.0 Matlab- 15.4	Dhaka- Male- 45 BMI >30 kg/m^2^-13.0 Daily smoker-6.0 Biomass user-4.0 Education >secondary-64.0 Monthly household income categories Lowest-5.0 Second-5.0 Third-17.0 Fourth-20.0 Fifth-19.0 Highest-34.0 Matlab- Male- 51% BMI >30 kg/m^2^-2.0 Daily smoker-9.0 Biomass user-98.0 Education >secondary-19.0 Monthly household income categories Lowest-20.0 Second-21.0 Third-29.0 Fourth-15.0 Fifth-9.0 Highest-6.0	Data was not separately presented for Bangladesh
Alim et al., 2014 [19]	Rural-Madla union, Bogra; Urban-Thanthania, Bogra;	Cross-sectional study	420	Non-pregnant, non-smoker, non-TB women aged 15 years or older having regular or daily cooking practice for at least 3 years	COPD	Chronic bronchitis- 8.0%; Severe bronchial obstruction based on PEFR-80.8%	Chronic bronchitis Biomass users-6.7 LP Gas users-1.5 Bronchial obstruction Biomass users mild-12.9 moderate-31.2 severe-55.4 Gas users mild-28.6 moderate-37.8 severe-29.1	Biomass user Chronic bronchitis OR: 5.94 95% CI, 1.02–34.45 p = 0.047 Severe bronchial obstruction OR: 4.54 95% CI, 2.10–9.82 p = 0.001
Mahfuz et al., 2014 [21]	Two major routes of Dhaka city (Mirpur to Mohakhali; Gabtoli to Mohakhali)	Cross-sectional	108	Helpers of human haulers having >6 months employment history in this position	Obstructive Pulmonary Impairment (reduced FEV_1_/FVC)	41.7	Not mentioned	Smoking OR: 3.62 95% CI, 1.87-4.67 Employment more than 24 months OR: 6.89 95% CI, 3.42-8.41
Biswas et al., 2016 [22]	Rural Chittagong	Cross- sectional observational study	276	Women >40 years	COPD	20.4	Age-specific 40-49 y- 9.8 50-59 y- 29.4 60-69 y- 51.0 >70 y- 9.8 Biomass user Biomass users- 16.4 Natural gas users-4 Life time smoking history Present - 6.4 Absent - 14.01 Tobacco chewing habit Present - 16.0 Absent - 4.4 Nature of kitchen Open -14.3 Closed - 23.9 Type of stove Outdoor -1.2 Indoor - 19.2 Seasonal variation in cooking Seasonal- 12.3 Always-23.7	Biomass fuel user OR: 3.4 95% CI, 1.6-7.14 p = 0.02 Tobacco chewing habit OR: 12.9 95% CI, 3.4-49.4 p = 0.001
Islam et al., 2013 [24]	Urban (Dhaka city)	Cross-sectional population-based survey	900	Male and female of age >35 years	COPD	11.4 (GOLD criteria)	Sex Male-11.7 Female- 10.6 Socio-economic status High-9.8 Low- 13.6	Increased age: p < 0.001) Male: p < 0.05 Smoker: p < 0.001 Low BMI: p < 0.05 Low SES: p < 0.05

According to the high-quality studies, the prevalence of COPD was greater among males and ranged from 11.7% to 22.0% compared to females (range: 6.4% to 10.6%) [17,24]. These two studies also reported that COPD was more prevalent among the low socio-economic groups (13.6% to 16.3%) than the high socio-economic groups (9.8% to 11.1%) [17,24]. Interestingly, Islam et al. 2013 found that the mean age of COPD patients was higher than that of the non-COPD patients (COPD: 57 years; non-COPD: 44 years) [24]. The pooled prevalence of COPD among people in the 40-49 years age group was 7.5% (range: 5.2% to 9.8%). On the other hand, this prevalence was 21.5% (range: 13.6% to 29.4%) among 50-59 years age group and 39.3% (27.5% to 51.0%) among people of 60-69 years age group [17,22]. A high-quality study conducted by Alam et al. (2015) reported that COPD was more prevalent among rural people (17.0%) than their urban counterparts (9.9%) [17]. The prevalence of COPD was also higher among biomass fuel users and ranged from 16.4% to 17.3% compared to clean fuel (e.g. LP gas or natural gas) users (range: 4.4% to 10.0%) [22,24]. Based on peak expiratory flow rate (PEFR)%, severe bronchial obstruction was also found higher among biomass fuel users (55.4 %) than gas users (29.1%) [17,19].

Risk Factors

For this review, we considered only high- and moderate-quality studies to identify the risk factors [17-19,21-22]. It should be noted here that one moderate-quality study was not considered here because it was a multicounty study and risk factors of COPD were not separately identified for Bangladesh [24]. All of these studies were conducted using cross-sectional design; however, the characteristics of study participants were distinctive. Data was analyzed following different statistical methods to identify risk factors like bivariate analysis (Pearson chi-square test and t-test), binary logistic regression [21], multivariate logistic regression [19,22,24], and conditional logistic regression [17]. However, only two studies adjusted potential confounders during analysis [17,19] (Table 2).

Tobacco Consumption

Tobacco consumption, in the form of smoking or chewing, was found associated with an increased prevalence of COPD in four studies [17,21-22,24]. One high-quality study reported that both current smokers (OR: 5.5; 95% CI, 4.2-7.2; *p* < 0.001) and former smokers (OR: 4.5; 95% CI, 3.3-6.0; *p* < 0.001) were at a higher risk to experience COPD after adjusting potential confounders [17]. Biswas et al. 2016 found that rural women (>40 years old) having tobacco chewing habit were 13 times more likely to suffer from COPD (OR: 12.9; 95% CI, 3.4-49.4; *p* < 0.001) [22].

Biomass Fuel Use

A significant association was reported with the development of COPD and the use of biomass fuel for cooking purpose in three studies [17,19,22]. Two moderate-quality studies found that biomass fuel use was positively associated with a higher prevalence of COPD among rural women [19,22]. A high-quality study that collected data from nearly 4,000 urban and rural participants also found that biomass fuel users were six times more likely to have COPD compared to clean fuel users (OR: 5.9; 95% CI, 1.0-34.5; *p* = 0.047) [17].

Old Age

Old age was reported as a risk factor for COPD by two studies [17,24]. Alam et al. (2015) observed that 50-59 years-aged people and 60-69 years-aged people were two times and five times more likely to develop COPD, respectively, compared to 40-49 years-aged people (50 to 59 years, OR: 2.2, 95% CI, 1.6-3.0; *p* < 0.001; 60 to 69 years, OR: 4.7, 95% CI, 3.5-6.4; *p* < 0.001) [17].

History of Asthma

A high-quality study reported that individuals with a history of asthma had seven times higher probability of developing COPD, even after adjusting for smoking history and other potential confounders (OR: 6.9; 95% CI, 4.9-9.5; *p* < 0.001) [17].

Others

Male gender, low socio-economic status, low body mass index (BMI), and long-time exposure to the hazardous environment as a result of longer employment history (helpers of human haulers) were also identified as risk factors of COPD in some studies; however, evidence of statistical significance was not clearly reported for these factors.

Discussion

To the best of our knowledge, this is the first systematic review that reported the prevalence and risk factors of COPD in Bangladesh. Although several prevalence studies have been conducted on COPD in Bangladesh, there is a scarcity of large-scale studies like nationwide COPD survey. This systematic review reported that the overall prevalence of COPD among Bangladeshi adults was 12.5% using the GOLD criteria. This finding is in accordance with the finding of a recent systematic review on the global and regional prevalence of COPD where it was reported that globally 11.7% individuals are currently suffering from COPD [7]. However, our data showed a higher prevalence of COPD in comparison to that in South East Asia (9.7%) [7]. One potential reason for this discrepancy might be studies included in the global and regional prevalence of COPD study were published between January 1990 and December 2014 [7]; however, studies included in our systematic review were published in or after 2013. COPD has the potential to impose a substantial burden on the health system of Bangladesh because of its high burden attributable to the exposure of its inhabitants to a wide range of behavioral (smoking) and environmental (air pollution) risk factors [27-28]. COPD is one of the leading but under-reported causes of mortality and morbidity in all over the world [29]. In Bangladesh, government and non-government organizations have been designing and implementing public health programs to combat NCDs such as diabetes and cardiovascular diseases, however, no such public health program is prevailing directed to COPD [30]. Public hospitals are not equipped enough to manage complicated cases of COPD [31]. Therefore, this is a timely need for policy-makers and public health professionals of Bangladesh to prioritize COPD. Nationwide survey is also warranted to identify the actual burden of COPD among diverse groups of populations across different geographical areas in Bangladesh in order to set effective and target specific public health intervention for prevention and control of this disease.

We were not able to find any study that could provide strong evidence on risk factors of COPD using longitudinal study design (cohort or case-control) or nationally representative sample. This result brings attention to the lack of availability of good-quality NCD-related data in developing countries as stated in a previous study [32]. Along with the scarcity of data, low quality of the existing literature accompanied by the inconsistency in the study site, study population, and statistical analysis impeded the comprehensive assessments of published articles in order to draw persuasive conclusions regarding risk factors of COPD in Bangladesh.

However, it was revealed from the existing literature that there is a strong association between tobacco use and COPD. Tobacco consumption, especially smoking, is established as one of the most important risk factors of COPD in all over the world [33]. Studies indicated that nearly half of the individuals with smoking habit develop COPD at any stage of their lives [34]. It is quite alarming that, in Bangladesh, 46.5% urban people and 55.5% rural people consume tobacco either by smoking or in a smokeless form [35]. It should be noted here that Bangladesh is a party of the WHO Framework Convention on Tobacco Control (FCTC) and developed several strong anti-tobacco policies and laws in last decade (e.g. prohibition of smoking at public place; restriction on tobacco advertising, promotion and sponsorship; obligation to cover at least 50% of major display area of tobacco products with visual presentation of adverse health impact of tobacco consumption) [36-37]. However, this high prevalence of tobacco consumption in Bangladesh indicates that there is a gap in law reinforcement and implementation of tobacco control programs. Therefore, further research is required to identify the facilitators and implementation challenges of tobacco control programs and laws in order to execute these programs in a more successful manner. Raising awareness about health impact of tobacco consumption using mass media and national education system can also be an effective approach to address this issue.

Our study identified biomass fuel use as a risk factor for developing COPD specifically among rural women. This finding is consistent with the result of other studies, which reported that biomass smoke exposure significantly increases the risk of developing COPD, especially in women [33,38]. A recent systematic review and meta-analysis stated that biomass fuel exposure is associated with COPD in different parts of the world [39]. A recent retrospective cohort study in Bangladesh also found that mortality and morbidity from lung diseases like COPD and chronic bronchitis are higher among biomass fuel users in comparison to that found among clean fuel users [40]. Biomass is presented in different forms, e.g. dried leaves, crop residues, shell, and coir of coconut, wood, sawdust, animal dung, coal, and used as a fuel for cooking and heating nearly by one-third of the global population [41]. Biomass is also the most commonly used cooking fuel in Bangladesh [42]. A recent study in China has shown that 50% reduction of solid-fuel use has the potential to reduce annual COPD death by 12.0% (4.3 million) for women [43]. Hence, to reduce biomass use-related COPD burden, clean fuel supply needs to be ensured at rural households in Bangladesh. Commencing newer technology like improved cooking stoves (to reduce indoor air pollution by increasing the efficiency of solid fuel) [44] or clean energy technology (to convert solid fuel directly into electrical work without combustion) [45] can also be an alternative in this regard. It might be challenging to introduce newer technology or guarantee the cost-effective and continuous supply of clean fuels in resource-poor settings of rural Bangladesh. Policy makers and implementers, therefore, can consider the experience of developing countries like India, Ghana, and Ethiopia [46-47], where these technologies have already been implemented.

Older age was identified as an important risk factor for developing COPD by some of the studies. It is well established that COPD prevalence, mortality, and morbidity increase with age [33]. Like other developing nations, Bangladesh is going through demographic transition triggering the figure of older population at the national level [48]. In 2010, 6.8% of the total country population was in the older age group (60 years and above). This group of people is estimated to comprise nearly one-tenth (11.7%) and one-fourth (22.3%) of the country population by the year 2020 and 2050, respectively [49]. As a result, a huge number of individuals will be at risk of suffering from different chronic diseases like COPD in the future. In the context of Bangladesh, elderly people are greatly dependent on others for their basic needs like food, shelter and health care [50]. Therefore, it is necessary for the pertinent stakeholders to develop appropriate policies and programmes in order to provide quality health care for elderly people suffering from COPD by ensuring financial solvency (by increasing work opportunity, providing old age allowance and retirement benefits); promoting social security and supporting health system preparedness considering the fact that good health of senior citizens of a country has the potential to contribute to the country’s economic growth by sharing their skill and experience.

## Conclusions

Currently, 12.5% of Bangladeshi adults are suffering from COPD in Bangladesh. This prevalence is much higher among tobacco users, biomass fuel users, and elderly people. As COPD is accountable for a substantial extent of mortality and morbidity, it is a timely need for policy makers and public health professionals of Bangladesh to prioritize COPD as an important health issue. Population awareness should be raised on health impact and economic burden of COPD by using mass media and including these issues in the national curriculum. Arranging policy dialogue is also imperative for promoting multi-sectoral response to combat COPD burden in Bangladesh.

## References

[1] American Thoracic Society (1995). Standard for the diagnosis and care of patients with chronic obstructive pulmonary disease. Am J Respir Crit Care Med.

[2] Laniado-Laborín R (2009). Smoking and chronic obstructive pulmonary disease (COPD). parallel epidemics of the 21st century. Int J Environ Res Public Health.

[3] Walker BR, Colledge NR (2018). Davidson's Principles and Practice of Medicine. Davidson's Principles and Practice.

[4] (2018). Chronic obstructive pulmonary disease (COPD). http://www.who.int/mediacentre/factsheets/fs315/en/.

[5] Soriano JB, Abajobir AA, Abate KH (2017). Global, regional, and national deaths, prevalence, disability-adjusted life years, and years lived with disability for chronic obstructive pulmonary disease and asthma, 1990-2015: a systematic analysis for the global burden of disease study 2015. Lancet Respir Med.

[6] McKay AJ, Mahesh PA, Fordham JZ, Majeed A (2012). Prevalence of COPD in India: a systematic review. Prim Care Respir Med.

[7] Adeloye D, Chua S, Lee C (2015). Global and regional estimates of COPD prevalence: systematic review and meta-analysis. J Glob Health.

[8] Ahsan Karar Z, Alam N, Kim Streatfield P (2009). Epidemiological transition in rural Bangladesh, 1986-2006. Glob Health Action.

[9] Biswas T, Islam MS, Linton N, Rawal LB (2016). Socio-economic inequality of chronic non-communicable diseases in Bangladesh. PLoS One.

[10] Islam AM, Majumder AA (2012). Hypertension in Bangladesh: a review. Indian Heart J.

[11] Saquib N, Saquib J, Ahmed T, Khanam MA, Cullen MR (2012). Cardiovascular diseases and type 2 diabetes in Bangladesh: a systematic review and meta-analysis of studies between 1995 and 2010. BMC Public Health.

[12] Sal-Sabil T, Islam A, Islam SS (2016). Risk factors for type 2 diabetes in Bangladesh: a systematic review. J Diabetol.

[13] Moher D, Liberati A, Tetzlaff J, Altman DG (2009). Preferred reporting items for systematic reviews and meta-analyses: the PRISMA statement. Ann Intern Med.

[14] Fowkes FG, Fulton PM (1991). Critical appraisal of published research: introductory guidelines. BMJ.

[15] Vandenbroucke JP, von Elm E, Altman DG (2007). Strengthening the reporting of observational studies in Epidemiology (STROBE): explanation and elaboration. PLoS Med.

[16] Sanderson S, Tatt ID, Higgins J (2007). Tools for assessing quality and susceptibility to bias in observational studies in epidemiology: a systematic review and annotated bibliography. Int J Epidemiol.

[17] Alam DS, Chowdhury MA, Siddiquee AT, Ahmed S, Clemens JD (2015). Prevalence and determinants of chronic obstructive pulmonary disease (COPD) in Bangladesh. COPD.

[18] Grigsby M, Siddharthan T, Chowdhury MA (2016). Socioeconomic status and COPD among low-and middle-income countries. Int J Chron Obstruct Pulmon Dis.

[19] Alim MA, Sarker MA, Selim S, Karim MR, Yoshida Y, Hamajima N (2014). Respiratory involvements among women exposed to the smoke of traditional biomass fuel and gas fuel in a district of Bangladesh. Environ Health Prev Med.

[20] Kabir MT, Basak D, Rahman MA (2016). The scenario of COPD in Dhaka city Bangladesh: extensive analysis of the prevalence, manifestations and standards of diagnosis and treatment. Int J Res Pharmacol Pharmacother.

[21] Mahfuz M, Ahmed T, Ahmad SA, Khan MH (2014). Altered pulmonary function among the transport workers in Dhaka city. Health.

[22] Biswas RS, Paul S, Rahaman MR (2016). Indoor biomass fuel smoke exposure as a risk factor for chronic obstructive pulmonary disease (COPD) for women of rural Bangladesh. Chattagram Maa-O-Shishu Hosp Med Col J.

[23] Rahman MM, Rahman MA, Sajani TT, Kawser A (2017). Prevalence of NCDs among rural households of Dhamrai Upazila, Dhaka. Anwer Khan Modern Med Col J.

[24] Islam MS, Hossain MM, Pasha MM, Azad AK, Murshed KM (2013). Prevalence and risk factors of chronic obstructive pulmonary disease (COPD) in Dhaka city population. Mymensingh Med J.

[25] Ahmed N (2016). Study on prevalence of asthma and COPD at Dhaka city in Bangladesh (doctoral dissertation, East West University). EWU Institutional Repository.

[26] Sterk PJ (2004). Let's not forget: the GOLD criteria for COPD are based on post-bronchodilator FEV1. Eur Respiratory Soc.

[27] Hossain S, Hossain S, Ahmed F, Islam R, Sikder T, Rahman A (2017). Prevalence of tobacco smoking and factors associated with the initiation of smoking among university students in Dhaka, Bangladesh. Cent Asian J Glob Health.

[28] Dasgupta S, Huq M, Khaliquzzaman M, Pandey K, Wheeler D (2006). Who suffers from indoor air pollution? evidence from Bangladesh. Health Policy Plan.

[29] Pauwels RA, Rabe KF (2004). Burden and clinical features of chronic obstructive pulmonary disease (COPD). Lancet.

[30] Bleich SN, Koehlmoos TL, Rashid M, Peters DH, Anderson G (2011). Noncommunicable chronic disease in Bangladesh: overview of existing programs and priorities going forward. Health policy.

[31] Zaman MM, Bhuiyan MR, Karim MN, Rahman MM, Akanda AW, Fernando T (2015). Clustering of non-communicable diseases risk factors in Bangladeshi adults: an analysis of STEPS survey 2013. BMC public health.

[32] Halbert RJ, Natoli JL, Gano A, Badamgarav E, Buist AS, Mannino DM (2006). Global burden of COPD: systematic review and meta-analysis. Eur Respir J.

[33] Mannino DM, Buist AS (2007). Global burden of COPD: risk factors, prevalence, and future trends. Lancet.

[34] Lundbäck B, Lindberg A, Lindström M (2003). Not 15 but 50% of smokers develop COPD?—report from the obstructive lung disease in Northern Sweden studies. Respir Med.

[35] Choudhury K, Hanifi SM, Mahmood SS, Bhuiya A (2007). Sociodemographic characteristics of tobacco consumers in a rural area of Bangladesh. J Health Popul Nutr.

[36] (2018). Legislation by country Bangladesh. https://www.tobaccocontrollaws.org/legislation/country/bangladesh/summary.

[37] (2018). National strategic plan of action for tobacco control, 2007-2010. Dhaka, Bangladesh: Ministry of Health and Family.

[38] Ekici A, Ekici M, Kurtipek E (2005). Obstructive airway diseases in women exposed to biomass smoke. Environ Res.

[39] Kurmi OP, Semple S, Simkhada P, Smith WC, Ayres JG (2010). COPD and chronic bronchitis risk of indoor air pollution from solid fuel: a systematic review and meta-analysis. Thorax.

[40] Alam DS, Chowdhury MA, Siddiquee AT (2012). Adult cardiopulmonary mortality and indoor air pollution: a 10-year retrospective cohort study in a low-income rural setting. Glob Heart.

[41] Fullerton DG, Bruce N, Gordon SB (2008). Indoor air pollution from biomass fuel smoke is a major health concern in the developing world. Trans R Soc Trop Med Hyg.

[42] Khan MN, Islam MM, Islam MR, Rahman MM (2017). Household air pollution from cooking and risk of adverse health and birth outcomes in Bangladesh: a nationwide population-based study. Environ Health.

[43] Lin HH, Murray M, Cohen T, Colijn C, Ezzati M (2008). Effects of smoking and solid-fuel use on COPD, lung cancer, and tuberculosis in China: a time-based, multiple risk factor, modelling study. Lancet.

[44] Jetter JJ, Kariher P (2009). Solid-fuel household cook stoves: characterization of performance and emissions. Biomass Bioenergy.

[45] Stambouli AB, Traversa E (2002). Solid oxide fuel cells (SOFCs): a review of an environmentally clean and efficient source of energy. Renew Sustain Energy Rev.

[46] Shrimali G, Slaski X, Thurber MC, Zerriffi H (2011). Improved stoves in India: a study of sustainable business models. Energy Policy.

[47] Pennise D, Brant S, Agbeve SM, Quaye W, Mengesha F, Tadele W, Wofchuck T (2009). Indoor air quality impacts of an improved wood stove in Ghana and an ethanol stove in Ethiopia. Energy Sustain Dev.

[48] Bairagi R, Datta AK (2001). Demographic transition in Bangladesh: what happened in the twentieth century and what will happen next?. Asia Pac Popul J.

[49] Islam MM (2016). Demographic transition and the emerging windows of opportunities and challenges in Bangladesh. J Popul Res.

[50] Kabir R, Kabir M, Uddin MS, Ferdous N, Chowdhury MR (2016). Elderly population growth in Bangladesh: preparedness in public and private sectors. IOSR J of Humanit Soc Sci.

